# Dichlorido[3-dimethyl­amino-*N*-(2-pyridylmethyl­ene)propyl­amine-κ^3^ *N*,*N*′,*N*′′]cadmium(II)

**DOI:** 10.1107/S1600536808035915

**Published:** 2008-11-08

**Authors:** Hong Lin, Xiao-Hong Geng, Yun-Long Feng

**Affiliations:** a Jinhua Professional Technical College, Jinhua, Zhejiang 321007, People’s Republic of China; b Zhejiang Key Laboratory for Reactive Chemistry on Solid Surfaces, Institute of Physical Chemistry, Zhejiang Normal University, Jinhua, Zhejiang 321004, People’s Republic of China

## Abstract

In the title mononuclear Cd(II) complex, [CdCl_2_(C_11_H_17_N_3_)], the Cd(II) atom is coordinated by two Cl atoms and three N atoms from the tridentate Schiff base ligand in a distorted square-pyramidal environment. The three N atoms and one Cl atom constitute the base of the pyramid, whereas the other Cl atom occupies the apical position.

## Related literature

For the properties of transition metal complexes with multidentate Schiff base ligands, see: Mukherjee *et al.* (2004 ▶); Saha *et al.* (2003 ▶). For Schiff base ligands derived from pyridine-2-carboxaldehyde and diamine acting as tridentate (NNN) ligands, see: Dalai *et al.* (2002 ▶); Mukherjee *et al.* (2001*a* ▶,*b* ▶). For the synthesis, see: Choudhury *et al.* (2001 ▶).
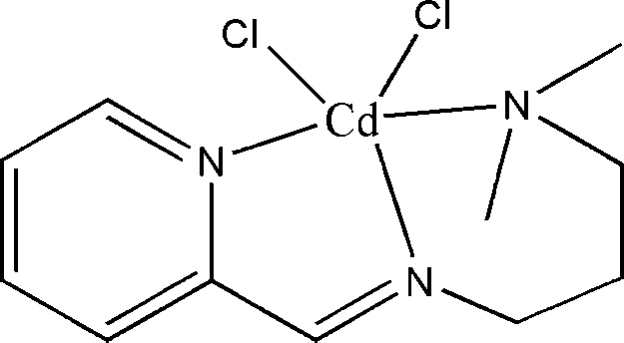


## Experimental

### 

#### Crystal data

[CdCl_2_(C_11_H_17_N_3_)]
*M* *_r_* = 374.59
Triclinic, 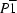

*a* = 7.6407 (15) Å
*b* = 9.0312 (18) Å
*c* = 11.860 (2) Å
α = 97.81 (3)°
β = 103.95 (3)°
γ = 111.11 (3)°
*V* = 718.2 (3) Å^3^
*Z* = 2
Mo *K*α radiation
μ = 1.88 mm^−1^
*T* = 293 (2) K
0.27 × 0.20 × 0.16 mm

#### Data collection

Bruker APEXII area-detector diffractometer
Absorption correction: multi-scan (*SADABS*; Sheldrick, 1996 ▶) *T*_min_ = 0.631, *T*_max_ = 0.753
12281 measured reflections
3251 independent reflections
3149 reflections with *I* > 2σ(*I*)
*R*_int_ = 0.020

#### Refinement

*R*[*F* ^2^ > 2σ(*F* ^2^)] = 0.018
*wR*(*F* ^2^) = 0.050
*S* = 1.14
3251 reflections
154 parameters
H-atom parameters constrained
Δρ_max_ = 0.33 e Å^−3^
Δρ_min_ = −0.72 e Å^−3^

### 

Data collection: *APEX2* (Bruker, 2004 ▶); cell refinement: *SAINT* (Bruker, 2004 ▶); data reduction: *SAINT*; program(s) used to solve structure: *SHELXS97* (Sheldrick, 2008 ▶); program(s) used to refine structure: *SHELXL97* (Sheldrick, 2008 ▶); molecular graphics: *SHELXTL* (Sheldrick, 2008 ▶); software used to prepare material for publication: *SHELXTL*.

## Supplementary Material

Crystal structure: contains datablocks I. DOI: 10.1107/S1600536808035915/at2652sup1.cif

Structure factors: contains datablocks I. DOI: 10.1107/S1600536808035915/at2652Isup2.hkl

Additional supplementary materials:  crystallographic information; 3D view; checkCIF report

## Figures and Tables

**Fig. 1.**
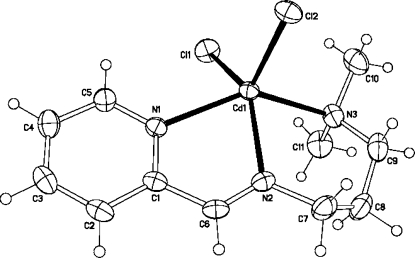
The molecular structure of (I), showing the atom-numbering scheme. Displacement ellipsoids are drawn at the 30% probability level.

## supplementary crystallographic information

### Comment

Transition metal complexes with multidentate Schiff base ligands have been extensively studied recently for their various crystallographic features, enzymatic reactions, catalysis, electrochemical and magnetic properties (Mukherjee *et al.*, 2004; Saha *et al.*, 2003). Literatures (Dalai *et al.*, 2002; Mukherjee *et al.*, 2001*a*,b) revealed that Schiff base ligands derived from pyridine-2-carboxaldehyde and diamine usually act tridentate (NNN) ones. The molecule of the title complex (I) (Fig.1) comprises one cadmium(II) ion, one neutral *N*-(pyridin-2-yl-methylene)-3-dimethylaminopropylamine ligand and two Cl^-^ ions. The Cd(II) atom is coordinated by two chlorine atoms and three nitrogen atoms from the tridentate ligand in a distorted square pyramidal environment. Four coordinated atoms of N(1), N(2), N(3) and Cl(1) constitute the base of the pyramid, whereas Cl(2) atom occupies the apical position.

### Experimental

The tridentate Schiff base, *N*-(pyridin-2-yl-methylene)-3-dimethylaminopropylamine (C_11_H_17_N_3_), were prepared by reflux of 0.5 mmol of 3-dimethylaminopropylamine and 0.5 mmol of pyridine-2-carboxaldehyde in 10 ml of ethanol for 30 min, according to the literature method (Choudhury, *et al.*, 2001). To 20 ml ethanolic and chloroformic solution (1:1) of the Schiff base (0.5 mmol), CdCl_2_.2.5H_2_O (0.5 mmol) in 5 ml water was added, with refluxing for 30 min. This mixture was cooled to room temperature and left to stand undisturbed. After 5 days colourless crystals (I) suitable for X-ray analysis were obtained.

### Refinement

The methyl groups were allowed to rotate to fit the electron density [C—H = 0.96 Å and *U*_iso_(H) = 1.5*U*_eq_(C)]; the other H atoms were positioned geometrically [aromatic C—H_aromatic_ 0.93 Å and aliphatic C—H = 0.97 Å, *U*_iso_(H) = 1.2*U*_eq_(C)].

### Crystal data

**Table:**

[Cd(C_11_H_17_N_3_)Cl_2_]	*Z* = 2
*M**_r_* = 374.59	*F*_000_ = 372
Triclinic, *P*1	*D*_x_ = 1.732 Mg m^−^^3^
Hall symbol: -P 1	Mo *K*α radiation λ = 0.71073 Å
*a* = 7.6407 (15) Å	Cell parameters from 3284 reflections
*b* = 9.0312 (18) Å	θ = 1.8–27.5º
*c* = 11.860 (2) Å	µ = 1.88 mm^−^^1^
α = 97.81 (3)º	*T* = 293 (2) K
β = 103.95 (3)º	Block, colourless
γ = 111.11 (3)º	0.27 × 0.20 × 0.16 mm
*V* = 718.2 (3) Å^3^	

### Data collection

**Table:**

Bruker APEX-II area-detector diffractometer	3251 independent reflections
Radiation source: fine-focus sealed tube	3149 reflections with *I* > 2σ(*I*)
Monochromator: graphite	*R*_int_ = 0.020
*T* = 293(2) K	θ_max_ = 27.5º
ω scans	θ_min_ = 1.8º
Absorption correction: multi-scan(SADABS; Sheldrick, 1996)	*h* = −9→9
*T*_min_ = 0.632, *T*_max_ = 0.754	*k* = −11→11
12281 measured reflections	*l* = −15→15

### Refinement

**Table:**

Refinement on *F*^2^	Secondary atom site location: difference Fourier map
Least-squares matrix: full	Hydrogen site location: inferred from neighbouring sites
*R*[*F*^2^ > 2σ(*F*^2^)] = 0.018	H-atom parameters constrained
*wR*(*F*^2^) = 0.050	*w* = 1/[σ^2^(*F*_o_^2^) + (0.0281*P*)^2^ + 0.1317*P*] where *P* = (*F*_o_^2^ + 2*F*_c_^2^)/3
*S* = 1.14	(Δ/σ)_max_ = 0.001
3251 reflections	Δρ_max_ = 0.33 e Å^−^^3^
154 parameters	Δρ_min_ = −0.72 e Å^−^^3^
Primary atom site location: structure-invariant direct methods	Extinction correction: none

### Special details

**Table:**

Geometry. All e.s.d.'s (except the e.s.d. in the dihedral angle between two l.s. planes) are estimated using the full covariance matrix. The cell e.s.d.'s are taken into account individually in the estimation of e.s.d.'s in distances, angles and torsion angles; correlations between e.s.d.'s in cell parameters are only used when they are defined by crystal symmetry. An approximate (isotropic) treatment of cell e.s.d.'s is used for estimating e.s.d.'s involving l.s. planes.
Refinement. Refinement of *F*^2^ against ALL reflections. The weighted *R*-factor *wR* and goodness of fit *S* are based on *F*^2^, conventional *R*-factors *R* are based on *F*, with *F* set to zero for negative *F*^2^. The threshold expression of *F*^2^ > σ(*F*^2^) is used only for calculating *R*-factors(gt) *etc*. and is not relevant to the choice of reflections for refinement. *R*-factors based on *F*^2^ are statistically about twice as large as those based on *F*, and *R*- factors based on ALL data will be even larger.

### Fractional atomic coordinates and isotropic or equivalent isotropic displacement parameters (Å^2^)

**Table:**

	*x*	*y*	*z*	*U*_iso_*/*U*_eq_	
Cd1	0.203590 (15)	0.391482 (13)	0.743794 (9)	0.03482 (5)	
Cl2	−0.08758 (6)	0.21422 (6)	0.57078 (4)	0.04931 (11)	
Cl1	0.15476 (8)	0.61916 (7)	0.85160 (5)	0.05592 (12)	
N2	0.4378 (2)	0.28768 (18)	0.72437 (13)	0.0403 (3)	
N3	0.1569 (2)	0.2335 (2)	0.88963 (13)	0.0475 (4)	
N1	0.4442 (2)	0.57453 (17)	0.67777 (13)	0.0367 (3)	
C1	0.5909 (2)	0.5312 (2)	0.66530 (14)	0.0374 (3)	
C2	0.7401 (3)	0.6249 (3)	0.62553 (16)	0.0472 (4)	
H2A	0.8408	0.5929	0.6189	0.057*	
C3	0.7372 (3)	0.7664 (2)	0.59584 (17)	0.0511 (5)	
H3A	0.8355	0.8310	0.5684	0.061*	
C4	0.5876 (3)	0.8108 (2)	0.60728 (17)	0.0508 (4)	
H4A	0.5829	0.9057	0.5873	0.061*	
C5	0.4430 (3)	0.7125 (2)	0.64904 (17)	0.0450 (4)	
H5A	0.3422	0.7435	0.6573	0.054*	
C6	0.5781 (3)	0.3739 (2)	0.69156 (15)	0.0422 (4)	
H6A	0.6750	0.3374	0.6838	0.051*	
C7	0.4187 (3)	0.1243 (2)	0.7381 (2)	0.0545 (5)	
H7A	0.5390	0.1123	0.7357	0.065*	
H7B	0.3109	0.0426	0.6712	0.065*	
C8	0.3807 (4)	0.0927 (3)	0.8542 (2)	0.0617 (6)	
H8A	0.3940	−0.0078	0.8648	0.074*	
H8B	0.4817	0.1811	0.9202	0.074*	
C9	0.1804 (4)	0.0784 (3)	0.8609 (2)	0.0611 (5)	
H9A	0.0825	0.0125	0.7845	0.073*	
H9B	0.1505	0.0192	0.9211	0.073*	
C10	−0.0528 (3)	0.1931 (3)	0.8813 (2)	0.0671 (6)	
H10A	−0.0874	0.1289	0.9371	0.101*	
H10B	−0.1360	0.1317	0.8014	0.101*	
H10C	−0.0707	0.2924	0.9000	0.101*	
C11	0.2795 (4)	0.3281 (3)	1.01250 (18)	0.0676 (6)	
H11A	0.2552	0.2592	1.0669	0.101*	
H11B	0.2471	0.4192	1.0334	0.101*	
H11C	0.4165	0.3676	1.0174	0.101*	

### Atomic displacement parameters (Å^2^)

**Table:**

	*U*^11^	*U*^22^	*U*^33^	*U*^12^	*U*^13^	*U*^23^
Cd1	0.03228 (8)	0.03880 (8)	0.03500 (8)	0.01555 (5)	0.01208 (5)	0.00852 (5)
Cl2	0.0374 (2)	0.0603 (3)	0.0398 (2)	0.01477 (19)	0.00686 (17)	0.00374 (19)
Cl1	0.0643 (3)	0.0572 (3)	0.0562 (3)	0.0338 (2)	0.0266 (2)	0.0059 (2)
N2	0.0416 (7)	0.0423 (7)	0.0395 (7)	0.0232 (6)	0.0094 (6)	0.0068 (6)
N3	0.0484 (8)	0.0496 (8)	0.0342 (7)	0.0104 (7)	0.0097 (6)	0.0114 (6)
N1	0.0354 (7)	0.0394 (7)	0.0366 (7)	0.0163 (6)	0.0137 (5)	0.0070 (6)
C1	0.0317 (7)	0.0468 (9)	0.0297 (7)	0.0156 (7)	0.0076 (6)	0.0024 (6)
C2	0.0326 (8)	0.0654 (12)	0.0386 (9)	0.0165 (8)	0.0120 (7)	0.0057 (8)
C3	0.0444 (9)	0.0539 (11)	0.0396 (9)	0.0032 (8)	0.0164 (8)	0.0050 (8)
C4	0.0613 (11)	0.0404 (9)	0.0455 (10)	0.0133 (8)	0.0205 (9)	0.0087 (7)
C5	0.0500 (10)	0.0421 (9)	0.0476 (9)	0.0212 (8)	0.0205 (8)	0.0101 (7)
C6	0.0379 (8)	0.0535 (10)	0.0401 (8)	0.0269 (8)	0.0111 (7)	0.0062 (7)
C7	0.0593 (12)	0.0429 (10)	0.0631 (12)	0.0289 (9)	0.0131 (10)	0.0075 (9)
C8	0.0744 (14)	0.0457 (10)	0.0634 (13)	0.0297 (10)	0.0078 (11)	0.0193 (9)
C9	0.0699 (14)	0.0435 (10)	0.0601 (12)	0.0121 (10)	0.0175 (11)	0.0188 (9)
C10	0.0568 (12)	0.0839 (16)	0.0576 (12)	0.0151 (11)	0.0277 (10)	0.0289 (12)
C11	0.0787 (16)	0.0705 (14)	0.0348 (10)	0.0200 (12)	0.0046 (10)	0.0076 (9)

### Geometric parameters (Å, °)

**Table:**

Cd1—N2	2.3418 (15)	C4—C5	1.390 (3)
Cd1—N1	2.3627 (16)	C4—H4A	0.9300
Cd1—N3	2.3992 (16)	C5—H5A	0.9300
Cd1—Cl2	2.4624 (15)	C6—H6A	0.9300
Cd1—Cl1	2.4637 (8)	C7—C8	1.517 (3)
N2—C6	1.260 (2)	C7—H7A	0.9700
N2—C7	1.465 (2)	C7—H7B	0.9700
N3—C11	1.469 (3)	C8—C9	1.512 (3)
N3—C9	1.480 (3)	C8—H8A	0.9700
N3—C10	1.484 (3)	C8—H8B	0.9700
N1—C5	1.338 (2)	C9—H9A	0.9700
N1—C1	1.346 (2)	C9—H9B	0.9700
C1—C2	1.382 (2)	C10—H10A	0.9600
C1—C6	1.470 (3)	C10—H10B	0.9600
C2—C3	1.378 (3)	C10—H10C	0.9600
C2—H2A	0.9300	C11—H11A	0.9600
C3—C4	1.370 (3)	C11—H11B	0.9600
C3—H3A	0.9300	C11—H11C	0.9600
			
N2—Cd1—N1	70.27 (5)	N1—C5—H5A	119.0
N2—Cd1—N3	84.79 (6)	C4—C5—H5A	119.0
N1—Cd1—N3	144.00 (6)	N2—C6—C1	120.98 (15)
N2—Cd1—Cl2	102.80 (4)	N2—C6—H6A	119.5
N1—Cd1—Cl2	109.74 (5)	C1—C6—H6A	119.5
N3—Cd1—Cl2	100.64 (5)	N2—C7—C8	112.77 (17)
N2—Cd1—Cl1	144.32 (5)	N2—C7—H7A	109.0
N1—Cd1—Cl1	91.30 (4)	C8—C7—H7A	109.0
N3—Cd1—Cl1	94.73 (5)	N2—C7—H7B	109.0
Cl2—Cd1—Cl1	112.28 (3)	C8—C7—H7B	109.0
C6—N2—C7	119.48 (16)	H7A—C7—H7B	107.8
C6—N2—Cd1	117.01 (12)	C9—C8—C7	114.81 (19)
C7—N2—Cd1	123.28 (12)	C9—C8—H8A	108.6
C11—N3—C9	110.90 (18)	C7—C8—H8A	108.6
C11—N3—C10	107.96 (18)	C9—C8—H8B	108.6
C9—N3—C10	108.30 (18)	C7—C8—H8B	108.6
C11—N3—Cd1	112.97 (13)	H8A—C8—H8B	107.5
C9—N3—Cd1	113.53 (12)	N3—C9—C8	116.67 (17)
C10—N3—Cd1	102.61 (13)	N3—C9—H9A	108.1
C5—N1—C1	118.26 (15)	C8—C9—H9A	108.1
C5—N1—Cd1	125.78 (12)	N3—C9—H9B	108.1
C1—N1—Cd1	115.93 (11)	C8—C9—H9B	108.1
N1—C1—C2	122.39 (17)	H9A—C9—H9B	107.3
N1—C1—C6	115.78 (15)	N3—C10—H10A	109.5
C2—C1—C6	121.77 (16)	N3—C10—H10B	109.5
C3—C2—C1	118.88 (18)	H10A—C10—H10B	109.5
C3—C2—H2A	120.6	N3—C10—H10C	109.5
C1—C2—H2A	120.6	H10A—C10—H10C	109.5
C4—C3—C2	119.15 (17)	H10B—C10—H10C	109.5
C4—C3—H3A	120.4	N3—C11—H11A	109.5
C2—C3—H3A	120.4	N3—C11—H11B	109.5
C3—C4—C5	119.26 (19)	H11A—C11—H11B	109.5
C3—C4—H4A	120.4	N3—C11—H11C	109.5
C5—C4—H4A	120.4	H11A—C11—H11C	109.5
N1—C5—C4	122.05 (18)	H11B—C11—H11C	109.5
			
N1—Cd1—N2—C6	1.15 (12)	N3—Cd1—N1—C1	47.48 (16)
N3—Cd1—N2—C6	−152.45 (14)	Cl2—Cd1—N1—C1	−98.36 (11)
Cl2—Cd1—N2—C6	107.80 (13)	Cl1—Cd1—N1—C1	147.36 (11)
Cl1—Cd1—N2—C6	−61.59 (16)	C5—N1—C1—C2	0.8 (2)
N1—Cd1—N2—C7	−173.34 (15)	Cd1—N1—C1—C2	178.90 (13)
N3—Cd1—N2—C7	33.06 (14)	C5—N1—C1—C6	−176.60 (15)
Cl2—Cd1—N2—C7	−66.69 (14)	Cd1—N1—C1—C6	1.52 (18)
Cl1—Cd1—N2—C7	123.92 (13)	N1—C1—C2—C3	−1.0 (3)
N2—Cd1—N3—C11	93.26 (16)	C6—C1—C2—C3	176.24 (16)
N1—Cd1—N3—C11	47.9 (2)	C1—C2—C3—C4	0.4 (3)
Cl2—Cd1—N3—C11	−164.66 (15)	C2—C3—C4—C5	0.3 (3)
Cl1—Cd1—N3—C11	−50.92 (16)	C1—N1—C5—C4	0.0 (3)
N2—Cd1—N3—C9	−34.13 (14)	Cd1—N1—C5—C4	−177.95 (14)
N1—Cd1—N3—C9	−79.53 (16)	C3—C4—C5—N1	−0.5 (3)
Cl2—Cd1—N3—C9	67.95 (14)	C7—N2—C6—C1	173.88 (16)
Cl1—Cd1—N3—C9	−178.31 (13)	Cd1—N2—C6—C1	−0.8 (2)
N2—Cd1—N3—C10	−150.77 (14)	N1—C1—C6—N2	−0.5 (2)
N1—Cd1—N3—C10	163.83 (13)	C2—C1—C6—N2	−177.89 (17)
Cl2—Cd1—N3—C10	−48.69 (14)	C6—N2—C7—C8	133.5 (2)
Cl1—Cd1—N3—C10	65.05 (14)	Cd1—N2—C7—C8	−52.1 (2)
N2—Cd1—N1—C5	176.55 (16)	N2—C7—C8—C9	68.8 (2)
N3—Cd1—N1—C5	−134.56 (15)	C11—N3—C9—C8	−66.7 (2)
Cl2—Cd1—N1—C5	79.59 (15)	C10—N3—C9—C8	175.00 (18)
Cl1—Cd1—N1—C5	−34.69 (14)	Cd1—N3—C9—C8	61.7 (2)
N2—Cd1—N1—C1	−1.40 (11)	C7—C8—C9—N3	−79.3 (2)
